# Is It Possible to Maintain High Compliance with the Enhanced Recovery after Surgery (ERAS) Protocol?—A Cohort Study of 400 Consecutive Colorectal Cancer Patients

**DOI:** 10.3390/jcm7110412

**Published:** 2018-11-04

**Authors:** Magdalena Pisarska, Natalia Gajewska, Piotr Małczak, Michał Wysocki, Piotr Major, Katarzyna Milian-Ciesielska, Andrzej Budzyński, Michał Pędziwiatr

**Affiliations:** 1 2nd Department of General Surgery, Jagiellonian University Medical College, Kopernika 21, 31-501 Kraków, Poland; magdalenapisarska@interia.pl (M.P.); natgajewska92@gmail.com (N.G.); pmmalczak@gmail.com (P.M.); michal92wysocki@gmail.com (M.W.); majorpiotr@gmail.com (P.M.); andrzej.budzynski@uj.edu.pl (A.B.); 2 Centre for Research, Training and Innovation in Surgery (CERTAIN Surgery), 31-501 Kraków, Poland; 3 Department of Pathology, Jagiellonian University Medical College, Kopernika 21, 31-501 Kraków, Poland; kmilianciesielska@gmail.com

**Keywords:** compliance with ERAS protocol, colorectal cancer, laparoscopy

## Abstract

The aim of our study was to evaluate the implementation and degree of adherence to the Enhanced Recovery after Surgery (ERAS) protocol in a group of 400 patients operated laparoscopically for colorectal cancer, and to assess its impact on the short-term results. The prospective study included patients with histologically confirmed colorectal cancer undergoing elective laparoscopic resection from years 2012 to 2017. For the purpose of further analysis, patients were divided into four groups: 100 consecutive patients were in each group. There were no statistically significant differences between groups in demographic parameters. The mean compliance with the ERAS protocol in the entire study group was 84.8%. Median adherence differed between the groups 76.9% vs. 92.3% vs. 84.6% vs. 84.6%, respectively (*p* < 0.0001). There were statistically significant differences between groups in the tolerance of oral diet (54% vs. 83% vs. 83% vs. 64%) and mobilization (74% vs. 92% vs. 91% vs. 94%) on the first postoperative day. In subsequent groups, time to first flatus decreased (2.5 vs. 2.1 vs. 2.0 vs. 1.7 days, *p* = 0.0001). There were no statistical differences in the postoperative morbidity rate between groups (*p* = 0.4649). The median length of hospital stay in groups was 5 vs. 4 vs. 4 vs. 4 days, respectively (*p* = 0.0025). Maintaining high compliance with the ERAS protocol is possible, despite the slight decrease that occurs within a few years after its implementation. This decrease in compliance does not affect short-term results, which are comparable to those shortly after overcoming the learning curve.

## 1. Introduction

Over the last two decades, modern perioperative care, known as the Enhanced Recovery after Surgery (ERAS) protocol, has been shown to enhance convalescence, reduce the length of hospital stay (LOS) and lower the complication rate in colorectal surgery [1,2,3,4,5]. It has also been reported that ERAS may influence long-term results [6,7,8]. It has been proven in numerous studies that greater adherence to the protocol is correlated with further improvement of the outcomes [9,10,11]. We have drawn the same conclusions in our previous works on ERAS compliance [12,13,14].

One of the elements of the ERAS protocol is the constant monitoring of protocol implementation among patients. There are concerns about the ability to maintain high compliance over a longer period of time. Some authors believe that it is not possible.

The aim of our study was to evaluate the implementation of the ERAS protocol in a group of 400 patients operated laparoscopically for colorectal cancer, and to assess its impact on the short-term results.

## 2. Materials and Methods

The prospective study included consecutive patients with histologically confirmed colorectal cancer undergoing elective laparoscopic resection from November 2012 to November 2017 at a tertiary referral university hospital. The annual volume of colorectal cancer patients in this centre is currently estimated at 80–120 cases. The surgery was performed by a laparoscopic surgeon with expertise in this type of surgery. 

In all patients, the 16-item ERAS protocol was applied as in our previous studies. The compliance with the protocol was calculated as the number of pre and intraoperative interventions fulfilled/13 (number of protocol elements included) as depicted in our previous studies [15]. The ERAS protocol was implemented in our unit in 2012 in all patients undergoing surgery for cancer [16]. Audit and outcome measures are performed every six months. We analysed compliance with the protocol among all members of the team (surgeons, anaesthetists, nurses, dieticians). Data from all the patients were prospectively recorded in a dedicated database. Following parameters were gathered and analysed: Sex, age, BMI (Body Mass Index), American Society of Anaesthesiologists (ASA) physical status, the presence of preoperative comorbidities, type of surgery and stage of cancer according to American Joint Committee on Cancer, operative time, intraoperative blood loss, morbidity, mortality, LOS and 30-day readmission rate. 

The work has been reported in line with the STROCSS (Strengthening the Reporting of Cohort Studies in Surgery) criteria [17].

### 2.1. Inclusion and Exclusion Criteria

**Figure 1:**
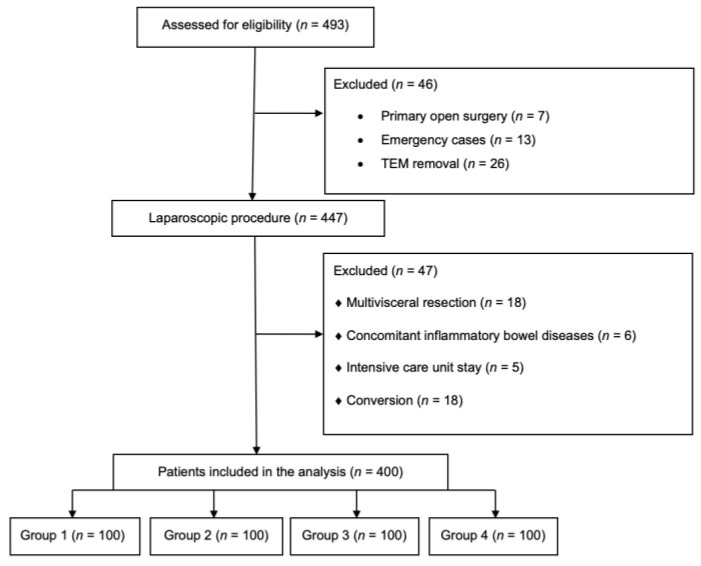
Patients flow through the study. TEM: transanal endoscopic microsurgery.

Inclusion criteria were: Histopathologically confirmed colorectal adenocarcinoma, laparoscopic resection of the colon and/or rectum, perioperative care based on the ERAS protocol. Exclusion criteria were: Initially open or emergency surgery, patients treated with transanal endoscopic microsurgery (TEM), multivisceral resection, conversion, concomitant inflammatory bowel disease, intensive care unit stay directly after surgery [18]. Figure 1 shows the patient flow through the study.

### 2.2. Statistical Analysis

All data were analysed with Statsoft Statistica v.13 (StatSoft Inc., Tulsa, OK, USA). The results are presented as mean ± standard deviation (SD), median with interquartile range (IQR) and odds ratios (OR) with 95% confidence intervals (CI) when appropriate. For the purpose of further analysis, patients were divided into four groups: 100 consecutive patients in each group. The study of categorical variables used the chi-square test of independence. The Shapiro-Wilk test was used to check for normal distribution of the data. In cases of quantitative variables, where no normal distribution was observed and when other requirements were not met, we used the Kruskal–Wallis test. In the case of fulfilling the requirements, we used the ANOVA test. For the analysis of specific sample pairs, post hoc tests were used. Additionally, a multivariate logistic regression analysis of parameters was undertaken to assess factors influencing occurrence of complications. The results were considered statistically significant when the *p*-value was found to be less than 0.05.

### 2.3. Ethical Approval

The study was approved by the local Ethics Review Committee (1072.6120.225.2017) and has been performed in accordance with the ethical standards laid down in the 1964 Declaration of Helsinki and its later amendments. Informed consent was obtained from all patients prior to surgery.

## 3. Results

Out of 493 patients with colorectal cancer operated in our department from November 2012 to November 2017, seven underwent primary open surgery, thirteen were submitted for emergency surgery and twenty-six underwent TEM resection. Additionally, patients with multivisceral resection (*n* = 18), concomitant inflammatory bowel disease (*n* = 6), those admitted to the intensive care unit after surgery (*n* = 5) and those requiring conversion (*n* = 18) were also excluded. Figure 1 shows the patient flow through the study.

### 3.1. Demographic Parameters

There were no statistically significant differences in demographic parameters such as sex, age, BMI, ASA scale, comorbidities, localisation and the stage of the tumor according to American Joint Committee on Cancer (AJCC) classification.

### 3.2. Operative Parameters

The groups differed in operative parameters. Median blood loss in groups 1 and 2 was 50 mL and in group 3 and 4–100 mL (*p* = 0.0002). Median operative time in groups 1, 2, 3 and 4 was 175 min, 210 min, 180 min and 185 min, respectively (*p* < 0.0001, significant difference between groups 1 and 2). Demographic and operative parameters are presented in Table 1.

### 3.3. Compliance with ERAS Protocol

**Figure 2:**
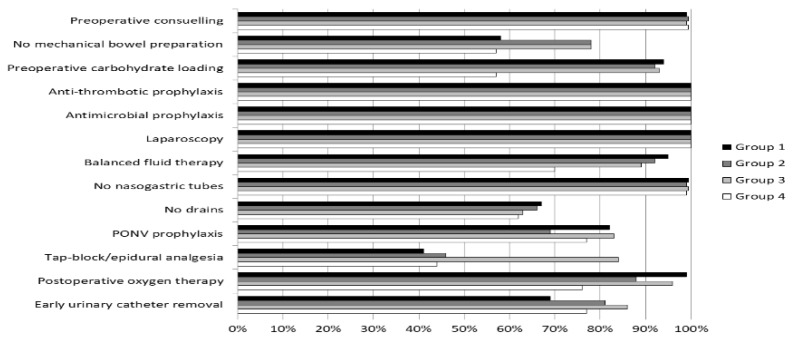
Compliance of the ERAS protocol elements in four groups. ERAS: Enhanced Recovery after Surgery; PONV: postoperative nausea and vomiting.

Compliance with the ERAS protocol elements in four groups are presented in Figure 2. The mean compliance with the ERAS protocol in the entire study group was 84.8% Median adherence differed between the groups, 76.9% vs. 92.3% vs. 84.6% vs. 84.6%, respectively (*p* < 0.0001) (Table 2).

### 3.4. Short-Term Outcomes

There were statistically significant differences between groups in the tolerance of the oral diet (54% vs. 83% vs. 83% vs. 64%, respectively) and mobilisation (74% vs. 92% vs. 91% vs. 94%, respectively) on the first postoperative day. In subsequent groups, time to first flatus decreased (2.5 vs. 2.1 vs. 2.0 vs. 1.7 days, *p* = 0.0001). There were no statistical differences in the postoperative morbidity rate between groups 1, 2, 3 and 4 (*p* = 0.4649) and its severity according to Clavien-Dindo classification (*p* = 0.4521). The median length of hospital stay in groups was 5 vs. 4 vs. 4 vs. 4 days, respectively (*p* = 0.0025) (Table 3). Readmission rate between groups was also comparable (11% vs. 10% vs. 8% vs. 12%, *p* = 0.7397).

### 3.5. Multivariate Analysis of Factors for Complications

Using multivariate regression model, we observed that only compliance with ERAS protocol (OR 1.81, 95% CI 1.13–2.88, *p* = 0.012) and type of surgery (rectal surgery) (OR 1.62, 95% CI 1.02–2.56, *p* = 0.041) were independent predictors of complications in our group. The remaining demographic parameters such as: Age, comorbidities and stage of disease had no influence on complications. 

## 4. Discussion

This study shows that despite the considerable motivation and efforts of the entire team to maintain high compliance with the ERAS protocol over time, thus sustaining faster recovery and improvement of short-term results (recovery parameters, LOS, complication rate), a small decline is observed. We noticed that there were differences in compliance for some of its elements over time. However, they did not affect short-term outcome such as complications, time to first flatus and LOS. The difference is, however, visible in the number of patients who entered the full oral diet on the first postoperative day and the opioids intake.

In our study, compliance with the ERAS protocol was high in all four analysed groups; however, it decreased in the third and stabilised in the fourth group of patients. The most significant differences in ERAS protocol compliance were in the first two analysed groups. These differences seem obvious, since the introduction of the ERAS protocol is a gradual process. The lowest compliance was in the first group, which is related to the process of implementation, as described in our previous works. We realised that a trained and motivated team is required for its implementation, which will then constantly monitor realisation of the protocol. In one of our previous works, we observed a rapid increase in compliance already after the first 30 patients (first six months after the introduction of ERAS to our unit) [19]. Other authors present a different number of patients in the learning curve, however, they also state that the highest compliance is achieved just after it has been exceeded. The same conclusions can also be drawn from this study. After a short phase of stabilisation, a decrease in implementation is observed, which results from the routine of applying ERAS protocol. Nevertheless, new employees are coming to each centre, hence the need to prepare appropriate educational materials, guidelines and conduct special training, especially for new residents. Most importantly high compliance requires full involvement of all members of the team who set their priorities for protocol items which are related to their specialty. A similar multidisciplinary approach has been described by Li et al. [20].

Interestingly, despite a slight decrease in ERAS compliance, we recorded a steady increase in the percentage of patients mobilised on the first postoperative day, which in the last group was as high as 94%. In addition, we observed shorter time to first flatus. Unfortunately, we noted a significant decrease in the percentage of successful introductions of the oral diet on the first postoperative day in the last group. Furthermore, we discovered that the latter group had a higher opioid intake. This interesting observation is quite difficult to comment on and most likely is related to the fact that this element of the ERAS protocol is not treated with sufficient attention and perhaps also to an aggressive approach to early mobilisation and the reduced use of TAP block analgesia in our unit.

A similar decrease in compliance with the ERAS protocol is also provided by other authors [21]. In one study, Martin et al. compare four consecutive years, first introduction and then three years after implementation of the ERAS protocol, showing a significant decrease in compliance in the last group, from 77% to 73% [22]. The decrease in compliance with the protocol was not related to worse clinical outcomes (functional recovery and 30-day complication rate). Also, the results presented by Gillissen et al., based on data from 33 Dutch hospitals with a follow-up of 3–5 years, showed stabilisation in the recovery function and the length of hospital stay, despite the decrease in compliance from 75% to 67% (difference was not statistically significant) [23]. Therefore, it seems that despite the initial decline in compliance, it is possible to maintain it at a stable level. This small decrease does not significantly affect convalescence and short-term treatment outcomes. In this study we also confirmed that low adherence to the protocol was an independent risk factor for complications. The relation between compliance and complications was confirmed with multivariate logistic regression analysis we performed. These results are in line with our previous articles. We have shown that better compliance with the ERAS protocol leads to better short-term observations [9,13]. The fact that small decrease in compliance does not significantly affect short-term outcomes might be explained by the hypothesis that, as the ERAS protocol continues, we subconsciously give up elements that seem less important to us. And even a slight decrease in the compliance does not worsen the results.

It is difficult to determine which elements of the ERAS protocol are the most difficult to maintain at a high level. In our study, both preoperative and intraoperative elements were kept constant regardless of the period in which they were analysed. A slight decrease in the compliance with the ERAS protocol resulted mainly from the postoperative elements. Similar results are presented by other authors [23,24,25]. Both the implementation and maintenance of postoperative elements is difficult. One such element is TAP block and epidural analgesia, and this element is dependent on anaesthesiologists. In our work, the biggest decrease in the last group concerned: TAP block, preoperative carbohydrate loading and balanced fluid therapy, which are elements dependent on anaesthesiologists. This phenomenon may be explained by the relatively high rotation among anaesthesiologists and the difficulty of keeping the training of all newcomers up to date. Multidisciplinary meetings and more rigorous monitoring of the ERAS elements should result in some improvement in the case in point. We also have to comment on the decrease in the number of patients with no mechanical bowel preparation in the last group, which results mainly from the recent increase in the number of patients treated with transanal total mesorectal excision (TaTME) techniques at our unit and changes in bowel preparation patterns [26]. Based on the results of the published work Garfinkle et al., we added oral antibiotics to preoperative preparation.

This study has limitations typical of a single centre study. To maintain a high number of patients enrolled in the study, we analysed both patients with colon and rectal cancer which may also create bias. The main limitation of this study is the lack of a control group—before ERAS implementation or group with very low compliance with the ERAS protocol. In our unit, the ERAS protocol is currently part of routine perioperative care independently of the type of surgery and operated organ; thus, the results cannot be generalised to different centres with worse protocol performance. Furthermore, the majority of the patients were operated laparoscopically (only a small percentage of conversions) and the data may not be comparable in a group of patients operated with an open approach. Additionally, we analysed only short-term outcomes—within 30 days after surgery. 

## 5. Conclusions

Maintaining a high compliance with the ERAS protocol is possible, despite the slight decrease that occurs within a few years after its implementation. This decrease in compliance does not affect short-term results, which are comparable to those shortly after overcoming the learning curve. The full implementation of the protocol is not possible for every patient, but it seems that it is possible to achieve good short-term results.

## Figures and Tables

**Table 1:** Demographic analysis of patient groups.

Parameter	Group 1	Group 2	Group 3	Group 4	*p* Value
Number of patients, *n*	100	100	100	100	-
Females, *n* (%)	47	48	39	48	0.5095
Males, *n* (%)	53	52	61	52	
Mean age, years ± SD	65.2 ± 12.2	64.2 ± 13.4	65.2 ± 13.5	62.4 ± 13.9	0.0712
BMI, kg/m^2^ ± SD	25.7 ± 4.4	26.9 ± 5.8	26.2 ± 4.3	26.7 ± 4.4	0.1102
ASA 1, *n* (%)	3	3	1	4	0.6989
ASA 2, *n* (%)	58	57	65	60	
ASA 3, *n* (%)	33	39	30	31	
ASA 4, *n* (%)	3	1	4	2	
Any comorbidity, *n* (%)	74	75	68	61	0.1167
Cardiovascular, *n* (%)	40	30	37	35	0.5061
Hypertension, *n* (%)	49	56	52	48	0.6699
Diabetes, *n* (%)	20	17	15	19	0.7962
Pulmonary disease, *n* (%)	15	14	5	7	
Renal disease, *n* (%)	6	11	5	5	0.3085
Liver disease, *n* (%)	3	5	5	3	0.7886
AJCC Stage 1, *n* (%)	27	44	36	36	0.1108
AJCC Stage 2, *n* (%)	37	27	25	27	
AJCC Stage 3, *n* (%)	24	20	21	29	
AJCC Stage 4, *n* (%)	12	9	18	8	
Colon, *n* (%)	67	74	59	61	0.1101
Rectum, *n* (%)	33	26	41	39	
Median operative time, min (IQR)	175 (130–200)	210 (180–240)	180 (150–240)	185 (150–220)	<0.0001
Median intraoperative blood loss, mL (IQR)	50 (30–100)	50 (50–100)	100 (50–200)	100 (50–150)	0.0002

SD: standard deviation; ASA: American Society of Anaesthesiology; AJCC: American Joint Committee on Cancer; IQR: interquartile range; BMI: Body Mass Index.

**Table 2:** ERAS protocol elements.

Parameter	Group 1	Group 2	Group 3	Group 4	*p* Value
Median compliance with ERAS protocol, % (IQR)	76.9 (69.2–84.6)	92.3 (84.6–100)	84.6 (76.9–92.3)	84.6 (76.9–92.3)	<0.0001 *
Preoperative compliance, % (IQR)	80 (60–100)	100 (90–100)	100 (80–100)	100 (80–100)	
Intraoperative compliance, % (IQR)	80 (60–80)	80 (80–100)	80 (60-80)	80 (60–80)	
Postoperative compliance, % (IQR)	67 (50–83)	83 (83.3–100)	83 (83–100)	83 (67–83)	
Introduction of full oral diet on the first postoperative day, *n* (%)	54	83	83	64	<0.0001 **
Mobilisation on the first postoperative day, *n* (%)	74	92	91	94	<0.0001*
No postoperative use of opioids, *n* (%)	58	67	58	44	0.0114 ***

* In post hoc analysis: only Group 1 differed from other Groups 2, 3, 4, ** In post hoc analysis: Group 1 differed from Groups 2, 3; Group 2 and 3 differed from Group 4, *** In post hoc analysis: Group 2 differed from Group 4. ERAS: Enhanced Recovery after Surgery; IQR: interquartile range.

**Table 3:** Postoperative outcomes in analysed groups.

Parameter	Group 1	Group 2	Group 3	Group 4	*p* Value
Time to first flatus, days ± SD	2.5 ± 1.4	2.1 ± 2.6	2.0 ± 1.8	1.7 ± 1.4	0.0001 *
Patients without complications, *n* (%)	67	77	73	73	0.4649
Patients with complications, *n* (%)	33	23	27	27	
Clavien-Dindo 1, *n* (%)	20	10	7	12	0.4521
Clavien-Dindo 2, *n* (%)	4	4	7	6	
Clavien-Dindo 3, *n* (%)	7	7	10	5	
Clavien-Dindo4, *n* (%)	1	2	1	3	
Clavien-Dindo 5, *n* (%)	1	0	2	1	
Mean length of hospital stay, days ± SD	6.6 ± 5.6	5.9 ± 6.2	4.9 ± 3.1	5.2 ± 3.4	0.0025 *
Median length of hospital stay, days (IQR)	5 (4–7)	4 (2–6)	4 (2–7)	4 (3–6)	
Readmission, *n* (%)	11	10	8	12	0.7397

* In post hoc analysis: only Group 1 differed from the other Groups 2, 3, 4. SD: standard deviation; IQR: interquartile range.
